# A18 EARLY LIFE EXPOSURE TO PARENTAL CROHN'S DISEASE IS ASSOCIATED WITH GUT MICROBIAL COMPOSITION AND FUNCTION, GUT PERMEABILITY AND INCREASED RISK OF FUTURE CROHN'S DISEASE

**DOI:** 10.1093/jcag/gwad061.018

**Published:** 2024-02-14

**Authors:** M Bushra, J Shao, L Qiu, P A Olivera, H Leibovitzh, M Xue, A Neustaeter, W Xu, O Espin-Garcia, G Aumais, H Huynh, R Panaccione, H Steinhart, M Cino, D Mack, J Marshall, M Ropeleski, A Bitton, K Jacobson, J Mcgrath, B Yerushalmi, M Abreu, C Bernstein, G Radford-Smith, C Lees, D Turner, M Silverberg, W Turpin, K Croitoru, S Lee

**Affiliations:** Faculty of Medicine, University of Toronto, Toronto, ON, Canada; lunenfeld-tanenbaum research institute, Mount Sinai Health System, Toronto, Toronto, ON, Canada; lunenfeld-tanenbaum research institute, Mount Sinai Health System, Toronto, Toronto, ON, Canada; Faculty of Medicine, University of Toronto, Toronto, ON, Canada; Faculty of Medicine, University of Toronto, Toronto, ON, Canada; lunenfeld-tanenbaum research institute, Mount Sinai Health System, Toronto, Toronto, ON, Canada; lunenfeld-tanenbaum research institute, Mount Sinai Health System, Toronto, Toronto, ON, Canada; University of Toronto Dalla Lana School of Public Health, Toronto, ON, Canada; Western University, London, ON, Canada; Hopital Maisonneuve-Rosemont, Montreal, QC, Canada; University of Alberta, Edmonton, AB, Canada; University of Calgary, Calgary, AB, Canada; lunenfeld-tanenbaum research institute, Mount Sinai Health System, Toronto, Toronto, ON, Canada; Faculty of Medicine, University of Toronto, Toronto, ON, Canada; Children's Hospital of Eastern Ontario, Ottawa, ON, Canada; McMaster University, Hamilton, ON, Canada; Queen's University, Kingston, ON, Canada; McGill University, Montreal, QC, Canada; The University of British Columbia, Vancouver, BC, Canada; Health Sciences Centre General Hospital, St. John's, , Canada; Ben-Gurion University of the Negev, Beer-Sheva, Southern, Israel; University of Miami School of Medicine, Miami, FL; University of Manitoba Max Rady College of Medicine, Winnipeg, MB, Canada; QIMR Berghofer Medical Research Institute, Herston, Queensland, Australia; Western General Hospital, Edinburgh, United Kingdom; Shaare Zedek Medical Center, Jerusalem, Jerusalem, Israel; lunenfeld-tanenbaum research institute, Mount Sinai Health System, Toronto, Toronto, ON, Canada; lunenfeld-tanenbaum research institute, Mount Sinai Health System, Toronto, Toronto, ON, Canada; Faculty of Medicine, University of Toronto, Toronto, ON, Canada; Faculty of Medicine, University of Toronto, Toronto, ON, Canada

## Abstract

**Background:**

Previous studies show that offspring of persons with Crohn’s disease (CD) have a 7-fold higher risk of incident CD than the general population. Newborns of women with CD (vs healthy women) have altered stool microbiome composition and elevated fecal calprotectin (FCP). The perinatal period (pregnancy and first year postpartum) is critical for the development of the gut microbiome and immune system.

**Aims:**

We investigated whether perinatal exposure to parental CD (compared to exposure later in life) has an impact on offspring’s gut barrier function, microbiome composition, and risk of future CD.

**Methods:**

We assessed 1252 healthy offspring of persons with CD who were recruited in the CCC-GEM Project. We classified offspring into "perinatally exposed (pregnancy and first year postpartum)" vs "exposed later in life" to parental CD diagnosis. We measured baseline FCP and urinary fractional excretion ratio of lactulose to mannitol (marker of intestinal permeability, LMR). Fecal microbiome composition was determined by 16S rRNA gene sequencing. Microbiome function was imputed by PICRUSt2 (q value defined as false discovery rate-adjusted p<0.05). Multivariable regression and Cox proportional-hazards analyses were used to assess if peri-natal exposure to parental CD is associated with LMR, FCP, altered microbial composition, and future CD onset.

**Results:**

Offspring exposed to a CD parent perinatally had a 4.7-fold higher risk (aHR 95%CI 1.3-17.5) of developing CD compared to those exposed to a parent who developed CD later in life (11/586 vs 3/666). LMR was significantly higher in the offspring exposed to parental CD perinatally (p=0.003), while no significant difference was observed for FCP at recruitment (p=0.94). Five bacterial taxa were significantly increased in the perinatally exposed group (q<0.05). The microbial functions such as glycine amidinotransferase, serine protein kinase and cyanophycin synthase were significantly increased in the perinatally exposed group (q<0.05).

**Conclusions:**

Offspring exposed to parental CD perinatally had a higher risk of developing CD than those exposed to parental CD later in life. Early life exposure to parental CD was associated with higher baseline LMR, altered bacterial taxa and functional capacities. Environmental exposure during the perinatal period may determine the offspring’s risk of developing CD.

**Funding Agencies:**

CAG, CCC, CIHRCanada Research Chair

